# Efficacy of Poly-3-Hydroxybutyrate Enriched with Simvastatin in Bone Regeneration after Tooth Extraction (Experimental Study)

**DOI:** 10.17691/stm2024.16.5.03

**Published:** 2024-10-30

**Authors:** K.M. Salekh, A.A. Muraev, A.A. Dolgalev, A.B. Dymnikov, G.A. Bonartseva, T.K. Makhina, D.V. Chesnokova, V.V. Voinova, A.P. Bonartsev, M.E. Mokrenko, S.Y. Ivanov

**Affiliations:** PhD Student, Department of Maxillofacial Surgery and Surgical Dentistry; The Patrice Lumumba Peoples’ Friendship University of Russia, 6 Miklukho-Maklaya St., Moscow, 117198, Russia; MD, DSc, Professor, Department of Maxillofacial Surgery and Surgical Dentistry; The Patrice Lumumba Peoples’ Friendship University of Russia, 6 Miklukho-Maklaya St., Moscow, 117198, Russia; MD, DSc, Professor, Department of Dentistry of General Practice; Stavropol State Medical University, 310 Mira St., Stavropol, 355017, Russia; MD, PhD, Associate Professor, Department of Maxillofacial Surgery and Surgical Dentistry; The Patrice Lumumba Peoples’ Friendship University of Russia, 6 Miklukho-Maklaya St., Moscow, 117198, Russia; PhD, Senior Researcher, A.N. Bakh Institute of Biochemistry; Federal Research Centre “Fundamentals of Biotechnology” of the Russian Academy of Sciences, 33/2 Leninsky Prospekt, Moscow, 119071, Russia; Researcher, A.N. Bakh Institute of Biochemistry; Federal Research Centre “Fundamentals of Biotechnology” of the Russian Academy of Sciences, 33/2 Leninsky Prospekt, Moscow, 119071, Russia; Junior Researcher, Department of Bioengineering, Faculty of Biology; Lomonosov Moscow State University, 1 Leninskiye Gory, Moscow, 119234, Russia; PhD, Senior Researcher, Department of Bioengineering, Faculty of Biology; Lomonosov Moscow State University, 1 Leninskiye Gory, Moscow, 119234, Russia; DSc, Associate Professor, Department of Bioengineering, Faculty of Biology; Lomonosov Moscow State University, 1 Leninskiye Gory, Moscow, 119234, Russia; PhD Student, Department of Maxillofacial Surgery and Surgical Dentistry; The Patrice Lumumba Peoples’ Friendship University of Russia, 6 Miklukho-Maklaya St., Moscow, 117198, Russia; MD, DSc, Professor, Corresponding Member of the Russian Academy of Sciences, Head of the Department of Maxillofacial Surgery and Surgical Dentistry; The Patrice Lumumba Peoples’ Friendship University of Russia, 6 Miklukho-Maklaya St., Moscow, 117198, Russia; Head of the Department of Maxillofacial Surgery; I.M. Sechenov First Moscow State Medical University (Sechenov University), 8/2 Trubetskaya St., Moscow, 119991, Russia

**Keywords:** bone plastic material, poly(3-hydroxybutyrate), simvastatin, porous microspheres, postextraction preservation, reparative osteogenesis, osteoinduction

## Abstract

Physiological resorption of bone tissue after tooth extraction leads to a decrease in the volume of bone tissue available for implantation and makes it difficult to install dental implants. Preservation of the well after tooth extraction is the solution to this problem, with the choice of bone plastic material playing an important role. The development of an “ideal” bone plastic material with osteoinductive properties that promotes reparative bone regeneration remains an urgent task.

**The aim of the study** was to evaluate the regeneration of bone tissue of the alveolar ridge during implantation of a new osteoinductive bone plastic material containing simvastatin into the wells of extracted teeth in sheep using microcomputer tomography.

**Materials and Methods:**

The study was conducted on 24 adult sheep with a total of 48 teeth removed. 12 wells were filled with material based on poly(3-hydroxybutyrate) (PHB) with simvastatin; 12 wells were filled with PHB-based material without simvastatin, 24 wells were used as a control. Micro-CT was used for comparative analysis of bone tissue formation between the test groups after 3 and 6 months.

**Results:**

The results of the study confirm the positive effect of simvastatin released from the PHB-based osteoplastic material on the volume of the formed bone tissue and the total bone volume in the defect area (BV/TV) and bone mineral density (BMD) 3 and 6 months after surgery.

**Conclusion:**

The study demonstrated that simvastatin, released from the PHB-based osteoplastic material, has an osteoinductive effect, promoting bone tissue regeneration in the wells left after tooth removal. Higher BV/TV and BMD values in the wells indicate better efficacy of the material in terms of regeneration support.

## Introduction

Tooth extraction is one of the most common surgical procedures in dentistry [1]. It is followed by inevitable physiological resorption of the bone tissue resulting in a decrease in bone volume in the alveolar process. This fact, in turn, complicates the subsequent placement of dental implants. This process is irreversible, and the only effective way to prevent bone loss is to preserve the well [2, 3]. Preservation is aimed at minimizing atrophies, maintaining the volume and shape of the alveolar process when preparing for dental implantation surgery [4].

It is known that preservation of the bone tissue volume is impacted both by preservation technique and the use of bone material with the required properties, such as biocompatibility, osteoconductivity, and osteoinductivity [5, 6]. Currently, there is no “ideal” bone material, thus the development of a bone plastic material with all major properties remains an acute activity in bone engineering [7, 8].

Poly-3-hydroxybutyrate (PHB) with simvastatin is an innovative bone material with unique properties, which is the reason for its perspective application in surgical dentistry and reconstructive surgery. This material combines several properties that ensure its biocompatibility, osteoinductivity, and osteoconductivity. The polymer base of the material (PHB) provides good biocompatibility, high levels of cellular adhesion, mechanical strength, and structural integration with the neighboring bone tissue [9]. Simvastatin, being part of the material, is an inhibitor of 3-hydroxy-3-methylglutarylcoenzyme A reductase, which promotes increased osteogenesis, inhibition of bone resorption, and anti-inflammatory response. This combined action of the polymer and simvastatin fosters effective regeneration of the bone tissue, which is of major importance in surgeries aimed at the alveolar process restoration [10].

Microcomputed tomography (micro-CT) is an X-ray imaging technique that allows studying the bone tissue at a microstructural scale with a resolution of 1 to 100 μm. Since its introduction into clinical practice, micro-CT has become an important tool for bone morphology analysis. In recent years, micro-CT has been widely used to assess changes in bone density and microarchitecture, dynamics of reparative osteogenesis, and research into new bone plastic materials, including in animal models [11, 12].

**The aim of this study** was to assess the effectiveness of the alveolar ridge bone regeneration stimulated by the application of a new bone plastic material based on poly-3-hydroxybutyrate and enriched with simvastatin during tooth extraction surgery in sheep using X-ray techniques.

## Materials and Methods

The *in vivo* study was conducted on 24 sheep of the North Caucasian meat-and-wool breed aged 24 months and weighing 65–70 kg. The experimental area was the sheep’s lower jaw with the first right and left premolars. The experimental study was based on the “split mouth” principle, when the defects of the wells of the extracted teeth on the left were experimental and divided into two groups (12 animals in each group) depending on the implanted material, whereas the defects of the wells of the extracted teeth on the right were used as a control. There were 48 teeth extracted in total. 12 wells were filled with the material based on porous PHB microspheres (obtained using a previously developed technique [13]) enriched with simvastatin (5%) [14] — test group 1; 12 wells were filled with the same material without simvastatin — test group 2; 24 wells were left for natural healing without bone materials — the control group. The animals were divided by the time of their withdrawal from the experiment — after 3 and 6 months: 6 sheep were selected from the test groups 1 and 2, 12 — from the control group.

All surgical interventions were made under general anesthesia with intramuscular administration of sodium thiopental. The dose was calculated in accordance with the manufacturer’s instructions and the animal’s body weight — 50 mg/kg. The animals were left unfed with free access to water for 24 h before the surgery. The following combination of drugs was used for premedication: droperidol 0.25% — 0.2 ml/kg of the body weight + relanium 0.5% — 0.2 ml/kg of the body weight + tramadol 1 ml intramuscularly. Anesthetized sheep were placed side-lying with fixation of the lower jaw. The area of the teeth for removal was antiseptically treated, the circular ligaments of the teeth were separated, and then the teeth were removed. In the test groups, the corresponding material was implanted into the wells and tightly fixed with resorbable sutures.

**Figure 1.**
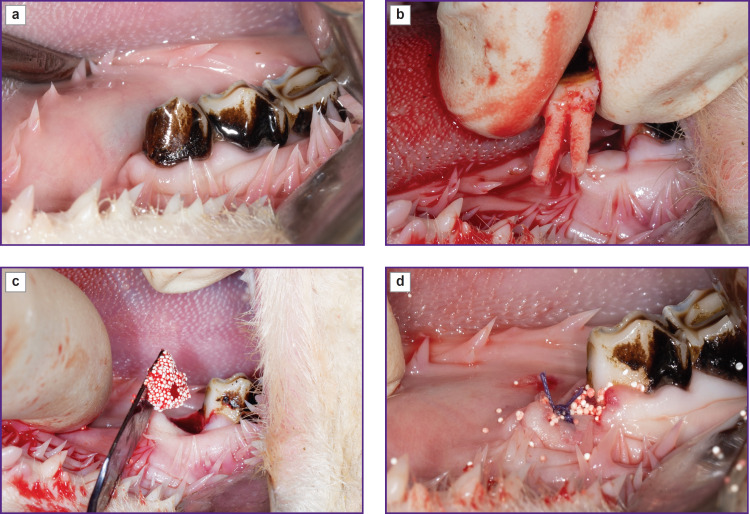
Stages of a tooth extraction followed by well preservation: (a) lower jaw of a sheep, third segment; (b) extraction of the 1^st^ premolar; (c) introduction of the PHB bone plastic material with simvastatin mixed with animal blood; (d) suturing of the well with resorbable sutures

The test material was pre-mixed with the animal’s blood before being put into the well of the extracted tooth, and thus its form became plastic, which eased its introduction into the well. The surgery scheme is shown in Figure 1.

All experiments were conducted in accordance with the guidelines and laws on animal protection and welfare as well as approved by the local ethics committee of the Patrice Lumumba Peoples’ Friendship University of Russia (Minutes of the Ethics Committee No.12 dated November 17, 2022). During the experiments, the requirements of regulatory and technical documents on the research with animals were strictly followed.

In the postoperative period, the animals were kept in an enclosure on a normal diet. After 3 and 6 months, respectively, they were euthanized by an overdose of a drug for general anesthesia of animals (Zoletil 100). A circular saw was used to isolate a bone area surrounding the initial surgical defect; the resulting samples were fixed in 10% buffered formalin solution for 72 h and then transferred to 70% isopropyl alcohol, where they were stored at 4°C until scanning.

Micro-CT examination of all prepared bone samples was performed using the X-ray micro-CT scanner Skyscan 1176, v. 10.0.0.0 (Bruker-microCT, Belgium) with the following pre-set parameters: X-ray voltage — 80–90 kV, current — 270–300 μA, Cu 0.1 mm and Cu+Al flters, image pixel size — 17.74 μm, tomographic rotation — 180–360º, rotation step — 0.2–0.3°.

The scanned objects were reconstructed using the NRecon software v. 1.7.4.2 (Bruker-microCT, Belgium) with the following main parameters: ring artifact reduction — 5–10, radiation quality — 41, minimum value for CS-to-image conversion — 0.002, maximum value for CS-to-image conversion — 0.037.

Analysis of the images for micro-CT structural and morphometric parameters was performed using the following software: CT-Analyser v. 1.18.4.0 (Bruker-microCT, Belgium) and CTVox v. 3.3.0r1403 (Bruker-microCT, Belgium).

Spatial orientation (x, y, z) and selection of specific areas of the reconstructed materials were performed in the DataViewer v. 1.5.6.2 software (Bruker-microCT, Belgium).

Bone mineral density specification, visualization, and data analysis were performed using the above-mentioned CT-Analyser v. 1.18.4.0 software.

When examining the well of the extracted tooth, the shape of the original defect was assessed; it was 5 mm wide, 8 mm long, and had a thickness corresponding to the distance from the lower edge of the enamel to the end of the root of the neighboring tooth.

The area of the bone defect was assessed by the ratio of the formed bone tissue volume and the total volume of the bone in the defect area (BV/TV, %), as well as by the bone mineral density (BMD, g/cm^3^), which was determined in various volumes of the bone isolated from samples.

### Statistical data processing

Data cleaning and descriptive statistics were performed in Microsoft Excel. The Shapiro–Wilk test, Student’s t-test for dependent samples, one-way analysis of variance, and Tukey’s procedure were performed using the following software packages: statsmodels v. 0.13.2, scipy v. 1.9.1, scikit_ posthocs v. 0.7.0, pandas v. 1.5.3, seaborn v. 0.11.2 of the Python programming language v. 3.9.13.

For each study group, the normality of data distribution was considered using the Shapiro–Wilk test. The sample mean and standard deviation (M±SD) were calculated for each group and each measurement (in Excel). In each group (test group 1, test group 2, control group), the measurement values after 3 and 6 months were intercompared using the Student’s t-test for dependent samples (the significance level α was equal to 0.05). The mean values in all groups were compared using one-way analysis of variance (ANOVA).

Taking into account that ANOVA resulted in statistically significant changes in at least one of the compared groups, a pairwise comparison of the groups was conducted using the Tukey procedure.

## Results and Discussion

Healing of the alveolar wells was uncomplicated in all animals. Three months after the material was sampled, the implant’s place in the experimental area (the third segment) was assessed and evidenced that the alveolar process had a smooth and even structure and demonstrated no visible signs of bone volume loss. However, in some control samples, crater-like retraction was seen in the well area.

**Figure 2.**
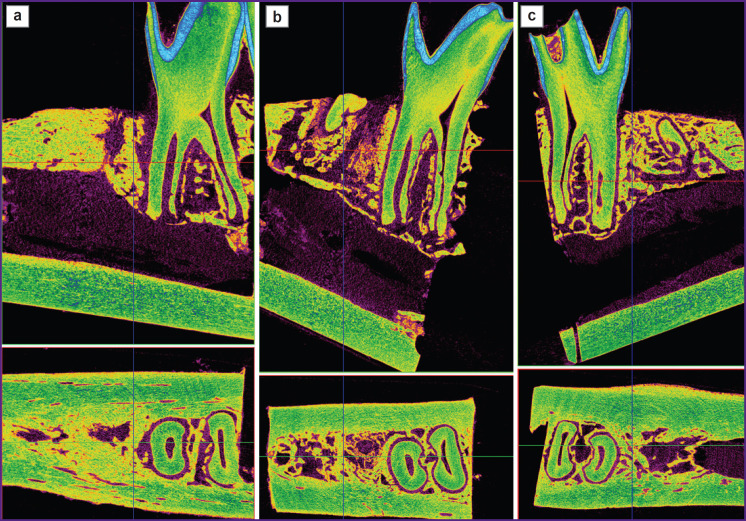
Micro-CT image of the extracted teeth wells 3 months after surgery: (a) test group 1 (PHB + simvastatin); (b) test group 2 (PHB); (c) control group

**Figure 3.**
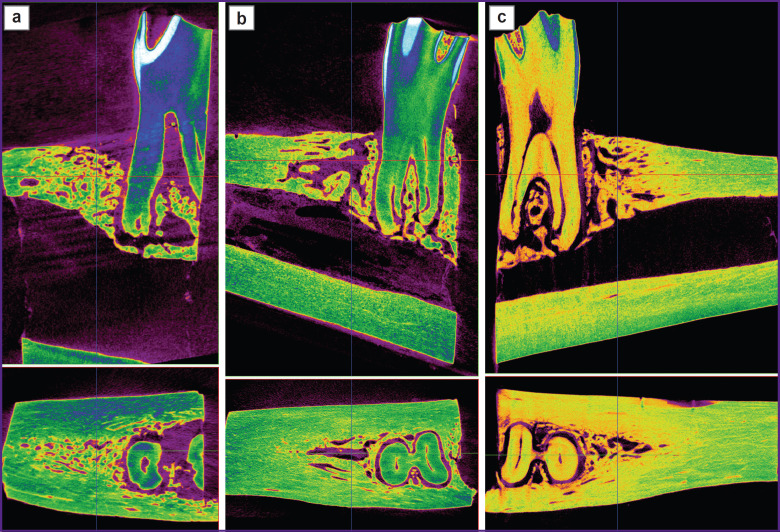
Micro-CT image of the extracted teeth wells 6 months after surgery: (a) test group 1 (PHB + simvastatin); (b) test group 2 (PHB); (c) control group

Images of the scanned samples from all three groups taken after 3 and 6 months are shown in Figures 2 and 3.

**Figure 4.**
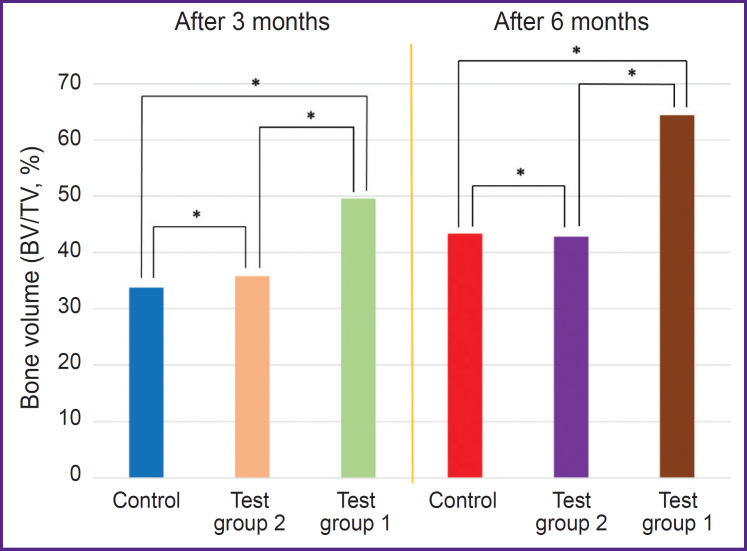
Changes in bone volume (BV/TV) in all study groups; * p<0.05

**Figure 5.**
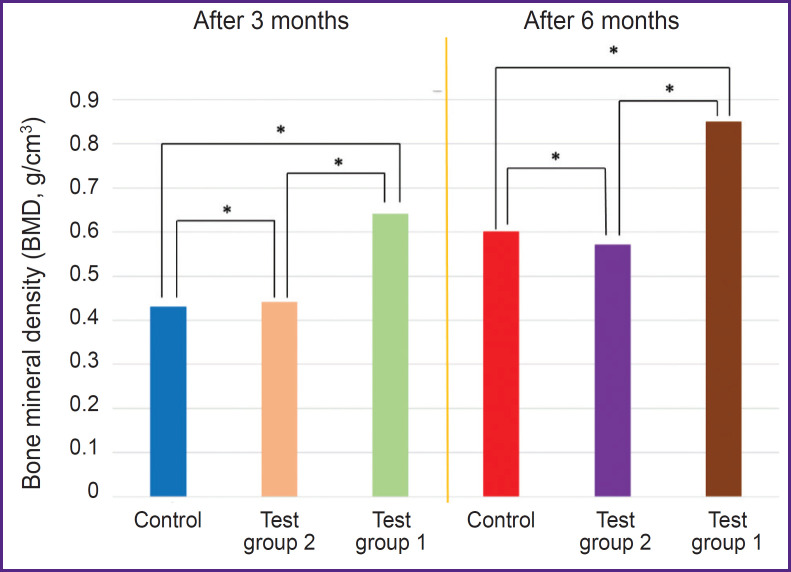
Changes in the bone mineral density (BMD) in all study groups; * p<0.05

Three months after the beginning of the experiment, an increase in all measured parameters was recorded in test group 1 (PHB + simvastatin) compared to test group 2 (PHB) and the control group. The BV/TV and BMD values in test group 1 (PHB + simvastatin) were significantly higher than in test group 2 (PHB). After 6 months, the mean values of these parameters changed again and demonstrated a significant increase in test group 1 and more moderate changes in test group 2 and the control group, thus proving the continued effect of simvastatin due to its prolonged release from the PHB-based material, which was verified earlier [15]. The results are shown in the Table and in Figures 4 and 5.

**Table:** Average values of the experiment’s results, M±SD

Group	3 months after surgery		6 months after surgery	
	BV/TV (%)	BMD (g/cm^3^)	BV/TV (%)	BMD (g/cm^3^)
Test group 1 (PHB + simvastatin)	49.44±3.48*^#^	0.64±0.05*^#^	64.35±1.44*^#^	0.85±0.01*^#^
Test group 2 (PHB)	35.77±2.12^#^	0.44±0.04^#^	42.67±4.61^#^	0.57±0.07^#^
Control group (unfilled well)	33.68±3.16	0.43±0.06	43.23±11.85	0.60±0.17

* statistically significant differences in values when comparing the test groups 1 and 2 with the control group; p<0.05;

^#^ when comparing the test groups 1 and 2 with each other; p<0.05.

In the wells filled with the PHB-based material releasing simvastatin, BV/TV and BMD had statistically significantly higher values: by 15.67% and 0.21 g/cm^3^, respectively, after 3 months and by 21.12% and 0.25 g/cm^3^, respectively, in 6 months after surgery compared to the control group, as well as an increase in the BV/TV and BMD values from month 3 to month 6 — by 14.91% and 0.21 g/cm^3^.

Therefore, the results of the analysis showed that 3 and 6 months after the tooth extraction surgery, a statistically significant difference in the BV/TV and BMD values was seen between animals from test group 1, which used PHB + simvastatin, and animals from test group 2 (PHB without simvastatin) and the control group.

## Conclusion

The study results confirmed the expressed osteoinductive effect of simvastatin released from the PHB-based osteoplastic material on the bone tissue regeneration in the wells after tooth extraction. The experimental data demonstrate a significant improvement in bone tissue parameters, such as BV/TV (bone volume to total volume) and BMD (bone mineral density), thus indicating high efficacy of the material in restoring the lost bone mass.

The perspectives of using this biomaterial in clinical practice, especially in dentistry, are very promising. Application of osteoplastic material with prolonged simvastatin release can be a significant breakthrough in preserving wells after tooth extraction, as its use allows to reduce the risk of bone resorption and provide optimal conditions for dental implantation.
